# Melt Temperature Estimation by Machine Learning Model Based on Energy Flow in Injection Molding

**DOI:** 10.3390/polym14245548

**Published:** 2022-12-18

**Authors:** Joohyeong Jeon, Byungohk Rhee, Jinsu Gim

**Affiliations:** 1 Department of Mechanical Engineering, Ajou University, Suwon 16499, Republic of Korea; 2 Wisconsin Institute for Discovery, University of Wisconsin-Madison, Madison, WI 53715, USA

**Keywords:** injection molding, melt temperature, temperature sensor, machine learning, melt temperature estimation

## Abstract

Highly reliable and accurate melt temperature measurements in the barrel are necessary for stable injection molding. Conventional sheath-type thermocouples are insufficiently responsive for measuring melt temperatures during molding. Herein, machine learning models were built to predict the melt temperature after plasticizing. To supply reliably labeled melt temperatures to the models, an optimized temperature sensor was developed. Based on measured high-quality temperature data, three machine learning models were built. The first model accepted process setting parameters as inputs and was built for comparisons with previous models. The second model accepted additional measured process parameters related to material energy flow during plasticizing. Finally, the third model included the specific heat and part weights reflecting the material energy, in addition to the features of the second model. Thus, the third model outperformed the others, and its loss decreased by more than 70%. Meanwhile, the coefficient of determination increased by about 0.5 more than those of the first model. To reduce the dataset size for new materials, a transfer learning model was built using the third model, which showed a high prediction performance and reliability with a smaller dataset. Additionally, the reliability of the input features to the machine learning models were evaluated by shapley additive explanations (SHAP) analysis.

## 1. Introduction

Injection molding technology is one of the mass-production manufacturing technologies for plastic-based materials, in which producers are required to achieve high product quality while maintaining a high level of reproducibility. The main process variables that affect product quality and reproducibility are the filling speed, packing pressure and time, cooling conditions, and melt temperature. Among these, melt temperature is an important factor that is deeply related to the rheological properties, especially viscosity [1]. It is often assumed that the melt temperature is similar to the barrel heater temperature, which is maintained at a constant value during the mold cycle. This assumption is used even in commercial injection molding computer-aided engineering (CAE) programs [2,3], wherein the melt temperature is commonly input as a constant value.

However, it has been reported that the temperature of the melt injected into a mold shows deviations from the barrel temperature and varies during an injection cycle. Amano and Utsugi measured the melt temperature in the barrel chamber and reported the qualitative relationships among the melt temperature and major plasticizing conditions, such as the rotational speed of the screw, the barrel temperature, and the back pressure [4,5]. In particular, the phenomenon and cause of melt temperature fluctuations during the injection cycle were investigated. Isayev and Hosaki measured the melt temperatures at various locations in the mold using thermocouples, from the filling to the ejection stages, to investigate melt temperature behaviors in the rubber molding process [6]. Although temperature measurements in the mold are advantageous for analyzing the melt temperature behaviors in the mold, they are not appropriate for quantifying the effects of the plasticizing conditions on the melt temperature, because the measured temperatures include the effects of the increase by compression and heat transfer to the mold. Jeon et al. measured the melt temperature profile using a relatively sensitive thermocouple sensor installed in the nozzle [7]; they reported that the melt temperature reacted to the plasticizing conditions, such as the screw rotational speed, and showed a large difference from the barrel temperature. Specifically, the melt temperature showed a large fluctuation of more than 25 °C under a specific condition. Numerical studies on the melt temperature have also been reported. Straka et al. developed a computational fluid dynamics model to explain the plasticizing process in the barrel [8]; they studied the thermal homogeneity of the melt from the calculated pressure and found that the melt temperature varied along the screw in a similar pattern to the results reported in the experimental study.

The misconceptions regarding the melt temperature may cause discrepancies between the designed and production results based on factors in the filling stage, such as melt viscosity, heat transfer to the mold, solidified layer formation, shear heating, and filling pattern; further, critical problems may also be caused in the production of precise products [9]. Zhou and Mallick reported that the melt temperature affected the orientations of anisotropic materials, which caused yield stress and fatigue strength of the products [10]. Wu and Liang reported that the melt temperature is related to the formation and size of the weldline [11]. Kamal et al. noted the melt temperature and pressure as the most important factors influencing the part weights and their corresponding built part weight prediction models [12]. Dubay et al. introduced a model predictive control approach to improve the melt quality by reducing the difference between the setting and the actual values [13]. According to previous research, it is necessary to comprehend the melt temperature to ensure a successful injection molding technology, because uncertainties regarding the melt temperature indicate uncertainties with respect to the part quality.

It is difficult to measure the temperature of the melt injected into a mold during the molding cycle. Polymeric materials have low thermal conductivities, so the temperature gradients of the materials are large. Further, these materials generate high shear stress in the flow owing to the high viscosity. The temperature sensors for the melt should endure temperatures of up to 300 °C and pressures of up to 100 MPa [14]. There are various available temperature sensors, such as thermocouples, thermistors, infrared sensors, and ultrasonic sensors. Among these, thermocouples are widely used owing to their low cost, wide temperature ranges, and short response times. Amano and Utsugi, who reported variations in the melt temperature, used a sheath-type thermocouple sensor in their experiments [4,5]. The sheath-type thermocouple is enclosed in a metallic protection tube, which protects the sensor from drift due to oxidation and low durability. However, the high thermal capacity of the sensor module, including the metallic tube, increases the response time. To enhance the response capabilities, thermocouples without the protection tube are often used. Sombatsompop et al. measured the radial temperature distribution in the nozzle with a thermocouple mesh [15]. Debey et al. designed film-type thermocouple sensors attached to thin plates to precisely measure the melt temperature behaviors in the mold [16].

Another method of obtaining fast measurement responses involves the use of infrared temperature sensors. Dontula et al. measured the melt temperature through an infrared sensor installed in the nozzle [17]; they reported that the melt temperature had a definite difference from the barrel temperature setting, and that this discrepancy was about 44 °C under specific conditions. Therefore, bare-wire thermocouples or infrared sensors provide high accuracies based on fast response times. However, bare-wire thermocouples are inadequate for the injection molding process in terms of durability and may hinder the melt flow to create shear heating and dead flow zones as invasive sensors. Infrared sensors also cause inconvenience when installed in the nozzle owing to their large size and difficulty in obtaining precise measurements due to differences in the opacity and emissivity of materials.

Ultrasonic temperature sensors are advantageous as noninvasive sensors. Praher et al. measured the radial distribution of the melt temperature in the barrel by installing many ultrasonic sensors on the barrel wall [18]. This method has the advantage of preventing some problems caused by invasive measurements. However, it incurs a relatively high cost, requires a special design to avoid the high-temperature environment of the injection molding process, and requires the complete pressure-volume-temperature (PvT) properties of the materials [19]. Additionally, it is difficult to apply this method to nonhomogeneous materials, such as foamed materials and fiber-/particle-reinforced composites. Therefore, each measurement method has advantages and disadvantages, so it is desirable to choose an appropriate method by considering the measurement purpose and the criteria.

As stated before, it is difficult to measure the melt temperature directly owing to many restrictions and requirements, which has motivated research to model and predict the melt temperature. Kamal and Kenig developed a theoretical model using the continuity, momentum, and energy equations for the major factors in the filling, packing, and cooling stages of the injection molding process [20]; their research was exploited to develop software to quantitatively analyze the injection molding process. However, there is a practical limit to building a governing equation for the melt temperature, because it is influenced by many factors. In this respect, machine learning models with artificial neural networks (ANNs) can be used as alternatives to the theoretical models. Zhao and Gao applied an ANN to predict the melt temperature profile [21]; they attempted to predict feature points, such as the maximum and minimum temperatures in the melt temperature profile, by considering that the profiles had regular shapes. Even if the predicted result followed the experimental data, a quantitative performance test of the model was not performed. Additionally, the model, built with only the process parameters, did not reflect the influences of the materials, environment, and injection machines.

ANNs have been applied to various aspects of the injection molding technology as well as melt temperature prediction. Lee et al. combined an ANN and a random search to develop a system for optimizing the process conditions of injection molding [22]. Tercan et al. attempted to minimize the discrepancy between the CAE analysis and experiments through a transfer learning model [23]. Gim et al. predicted the part weights using a machine learning model with in-mold pressure profiles, which was difficult for the operators to analyze [24]. Recently, new technological advances in machine learning and development of computing performance have rapidly increased to enable the building and application of machine learning models to predict many aspects of the injection molding technology, which is difficult with the theoretical models.

In this work, to ensure precise measurement of the melt temperature, a bare-wire thermocouple sensor with high accuracy and fast response time was developed. The sensor structure can withstand high temperatures and pressures during injection molding. Although the sensor was sensitive enough, it was not suitable for the actual environment of the injection molding process owing to limited durability and difficulty in operation. Therefore, three machine learning models were constructed based on the measured melt temperatures to predict the melt temperature profiles in the barrel during injection molding without the use of melt temperature sensors. The first model accepted the process setting parameters as the input features and was built for comparisons with previously reported models. With respect to the energy flow during the plasticizing process, major features such the energy consumption of the screw-driving motor and heat generation of the barrel heaters were selected as the input features to the second model. The process data under various conditions were acquired in the experiments. The third model included features related to the energy that the material gained, such as the specific heat and weight of the molded part. Compared to conventional models with only the process setting parameters, we attempted to reflect the formation process of the melt temperature more specifically through our machine learning model.

## 2. Method

For successful machine learning, the training dataset should be chosen such that it has an intimate relationship with the prediction problem [25]. In previous research on predicting the melt temperature during plasticizing, the process setting parameters such as the screw rotational speed, heater band temperatures, and the back pressure were used as training data [21]. The plasticizing process is also influenced by the state of the injection molding machine, environmental conditions, and material properties as well as process setting parameters. If the materials or the injection molding machines are changed, the machine learning model trained with only the process setting parameters should be trained with a new dataset, which is inefficient in terms of cost and time. To develop a machine learning model for the practical purpose of manufacturing, it is desirable to consider the cost and time of the training dataset.

**Figure 1:**
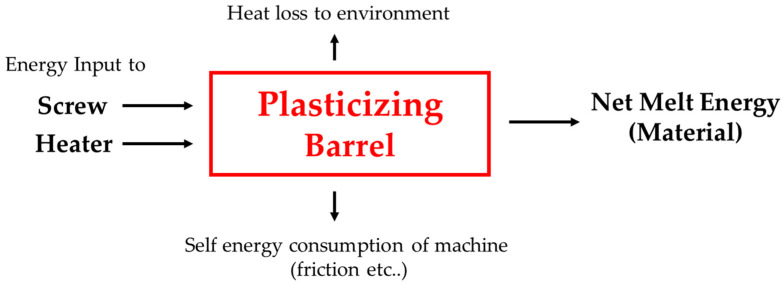
Energy input and loss in the injection molding machine.

Figure 1 shows the energy flow from the moment when a material is loaded into the hopper to the completion of plasticizing. The material is heated by shear heating owing to screw rotation and heat transfer from the barrel heaters. The heating and plasticizing processes depend upon the specifications of the injection molding machine such as the screw and heaters; material properties such as thermal conductivity and specific heat; and other factors such as the friction coefficient between the pellets and barrel surface as well as the size and shape of the pellets.

**Figure 2:**
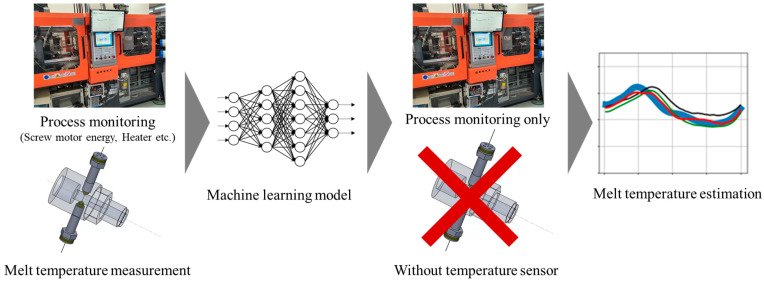
Schematic illustration of the proposed methodology.

Figure 2 shows the schematic of the methodology proposed in this study. To reflect the specific plasticizing process, the energies transferred by the injection molding machine to the material and environment were monitored. Through such monitoring, a training dataset consisting of features closely related with the forming process of the melt temperature profile was built. Additionally, reflecting the material properties and interactions with the injection molding machine could allow the machine learning model to achieve high accuracy with a limited dataset. Because the size of the training dataset is proportional to cost and time, higher productivity may be achieved by building a training dataset consisting of features closely related to the target process. The machine learning models trained with data acquired from the injection molding machine were developed to predict the melt temperature profile without a melt temperature sensor in the nozzle. It is desirable to exploit existing machine learning models when the injection molding machine and materials are changed. For this purpose, a transfer learning model was built to achieve high accuracy with a limited size of the new training dataset.

## 3. Experiments

### 3.1. Materials and Instruments

**Figure 3:**
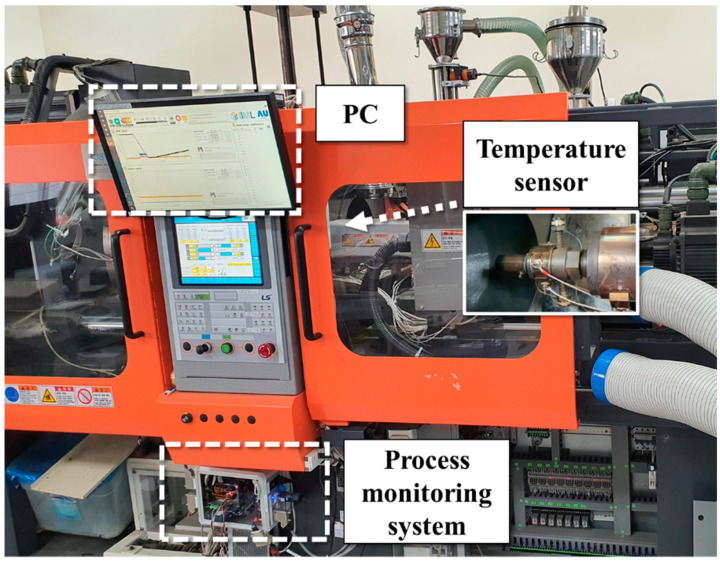
Measurement system.

Figure 3 shows the measurement system configuration used in this work. A 150-ton electric injection molding machine (LGE 150C by LS Mtron, Anyang, Republic of Korea) was used in the experiments. The nozzle adapter, including the melt temperature sensor, was installed between the nozzle and barrel. The signal from the thermocouple was conditioned and acquired using the thermocouple module (NI 9214 by National Instruments, Austin, TX, USA) at a sampling rate of 10 Hz. The speed seemed to be inadequate, but the filtering and noise reduction processes were completed at this speed, so it was deemed to be sufficient for the experiment. The power of each heater was measured with a current sensor (FS4C by Fine-trans, Incheon, Republic of Korea). The signals of the plasticizing motor power, screw position, and back pressure were supplied by the injection molding machine and acquired with a data acquisition device at 500 Hz sampling rate. The mold used in the experiment was designed to have a simple geometry so as to quickly transfer heat to the cooling channels. The mold design ensured quick solidification of the material and uniform cycle time. The materials used in the experiment were acrylonitrile butadiene styrene (ABS, HF380 by LG Chem., Seoul, Republic of Korea) and high density polyethylene (HDPE, JH910 by SK Chem., Seongnam, Republic of Korea).

### 3.2. Temperature Sensor

**Figure 4:**
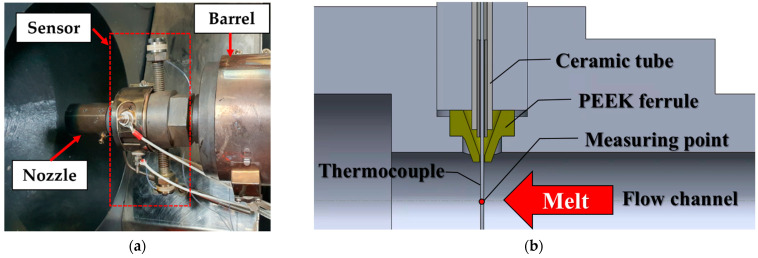
Melt temperature sensor: (**a**) temperature sensor mounted on the injection molding machine; (**b**) internal detailed structure.

The melt temperature sensor in the experiments should satisfy some requirements, such as durability and a sufficiently fast response time. The sheath-type thermocouple sensors were not adequate for this experiment because of their lengthy response times owing to the high heat capacity of the protection tube, even though they are widely used to measure the melt temperature in the nozzle. The bare-wire thermocouples used in the experiments may be destroyed by the high shear stress of the melt; therefore, a bare-wire k-type thermocouple of 0.5 mm diameter butt-welded in the middle (CHAL-020-BW by Omega Engineering, Biel/Bienne, Switzerland) was installed in the nozzle adapter, as shown in Figure 4a. Such a thin temperature sensor is advantageous not only in response time but also in measurement accuracy, because the heat transfer with the barrel wall is reduced [26]. As a result of performance tests, the time constant of the bare-wire thermocouples was 1/10 of the sheath thermocouples with a diameter of 3 mm. When fluid passed through at a constant temperature, it took 3.85 s to reach 63.2% of the steady state value with the sheath-type thermocouple, whereas the bare-wire thermocouple took 0.38 s.

The structure of the melt temperature sensor module is shown in Figure 4b. To prevent leakage through the installation hole of the sensor during injection molding at a high temperature and pressure, a ferrule made of polyether ether ketone (PEEK) was fabricated and installed; PEEK has a glass transition temperature of 140 °C and a melting temperature of 343 °C. The ferrule was designed to firmly grip the thermocouple wire by the pressure in the nozzle adapter for effective sealing. The thermocouple wire was insulated electrically and thermally by the ferrule and ceramic tube. The long guiding tube of the thermocouple wire with a tight clearance prevents possible accidental leakage by maintaining a low temperature. The tube was exposed to the atmosphere so that the melt in the gap of the tube would solidify quickly. The sensor developed in this work was durable without leakage after more than 30,000 cycles, including filling and packing at 60 MPa.

### 3.3. Energy Monitoring

The energy transferred to the material in the barrel comprises the mechanical energy by screw rotation and thermal energy by the barrel heaters, as shown in Equation (1).

(1) $$E_{Material}≅(E_{screw}-E_{machine})+(E_{heater}-E_{heat\_loss})$$

- $E_{material}$: internal energy change after the material is fed to the hopper before the melt is injected through the nozzle
- $E_{screw}$: energy transferred to the material by screw rotation
- $E_{machine}$: energy loss without being transmitted to the material, such as that from friction
- $E_{heater}$: energy supplied to the heaters in a cycle
- $E_{heat\_loss}$: heat energy loss by convection to the atmosphere and conduction to the machine

**Figure 5:**
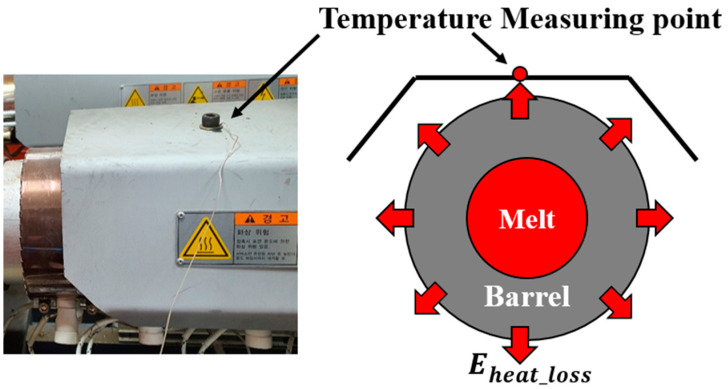
Temperature measurement at the top of the safety guard of the barrel to approximate the heat transfer rate to the surroundings.

*E_Material_* was calculated from the material temperature difference between the hopper and the nozzle as well as the specific heat data measured by a differential scanning calorimeter (DSC). *E_screw_* was calculated from the measured electrical energy supplied to the screw motor during plasticizing. *E_machine_* was the total energy loss in the injection molding machine caused by friction, motor efficiency, etc.; the energy loss was estimated by measuring the energy supplied to the screw motor when the screw rotated without a material in the barrel. *E_heater_* was acquired by measuring the electrical energy supplied to the barrel and nozzle heaters. It was difficult to measure *E_heat_loss_*; because it was relatively easy to measure the previous four energies, *E_heat_loss_* could be calculated indirectly. The atmospheric temperature closely related with *E_heat_loss_* was measured and supplied to the training dataset to estimate the approximate heat loss. Figure 5 shows the measurement location of the atmospheric temperature, which is the top surface of the barrel guard.

### 3.4. Experimental Conditions

**Figure 6:**
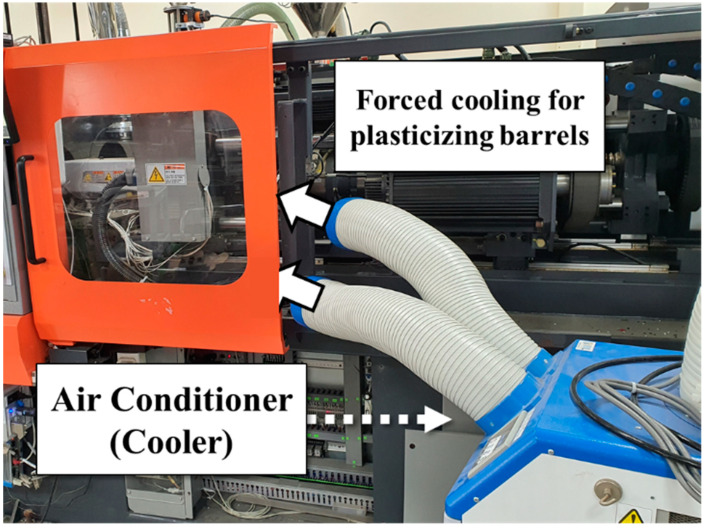
Forced cooling of the plasticizing barrel using a cooler; the input energy of the heater varies significantly.

The experimental conditions for the HDPE and ABS are listed in Table 1 and Table 2, respectively. The process variables that influence the plasticizing process as well as resident time and state of the melt in the barrel were selected. Experiments were performed by the orthogonal array of design of experiment (DOE). The flat condition in the heater profile indicates that all heaters of the barrel are set to the same temperature, whereas the decrease condition indicates that the heater temperatures decrease linearly toward the hopper and that the temperature of the hopper side heater is set to a value 40 °C lower than that of the nozzle heater. For the forced cooling condition, cold air was blown over the barrel heaters using an air conditioner, as shown in Figure 6, to change the convective heat transfer condition. The dwell time condition had the purpose of analyzing the effects of the staying time of the melt in the chamber before the start of injection after the completion of plasticizing. In the ABS experiment, the experiment was reduced to having two levels, instead of the three used in the HDPE experiment. This was intended to verify the prediction accuracy by transfer learning in the ABS experiment through a smaller dataset than the machine learning model trained with the dataset of the HDPE experiment.

The melt temperature was measured by advancing the screw at a speed of 10 mm/s to ensure that the melt flowed by the sensor in the nozzle adapter. It was important to maintain a uniform cycle time under a certain process condition, because the purpose of the experiment was to measure the heating history of the material from the moment of hopper loading to injection through the nozzle. Hence, the mold was designed to allow for rapid solidification of the material so as to not interrupt the cycle time. All experiments were conducted in the automatic cycle mode. The injection molding process requires a stabilization time of a certain cycle until the system reaches a steady state when the process conditions change [9]. For each experimental condition, the measured data from the first 15 trials after changing the experimental conditions were not collected, and the data of the subsequent 35 trials were collected and included in the training dataset. This allows for data to be collected after the process is as stabilized as possible. These 35 pieces of collected data were averaged and added to the dataset. Thus, the training dataset consisted of a total of 216 HDPE data points ($3^{3}\times 2^{3}$) and 64 ABS data points ($2^{6}$).

### 3.5. Machine Learning Model

**Figure 7:**
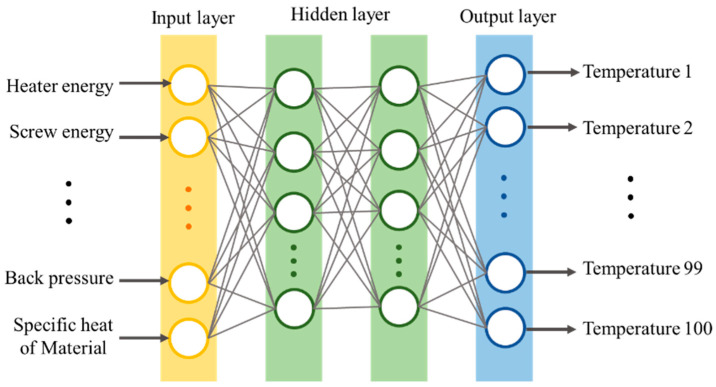
Multilayer neural network model structure used in the experiment.

The ANN was supplied the process setting values, measured data, and material properties in the input layer to obtain the melt temperature as the output layer, as shown in Figure 7. Owing to the large differences among the input data values, all data were normalized to enhance stability and accuracy. About 75% of the total dataset was chosen arbitrarily for training, and the rest was set as the validation dataset. The structure of the multilayer ANN is shown in Table 3; it was optimized using Optuna 3.0.4 (Preferred Networks, Tokyo, Japan), which is a hyperparameter optimization framework [27]. To evaluate the prediction performance of the machine learning model proposed in this work, three models trained with different datasets were evaluated. Model 1 was trained with a dataset comprising the process setting parameters only. Model 2 was trained with a dataset comprising the process parameters measured by the monitoring system as well as the data of Model 1. Model 3 was trained with a dataset containing all the data of Model 2 and additionally with the material properties. The input features for each model are listed in Table 4. All neural network configuration and training was performed using Tensorflow 2.9.1. (Google Brain, Mountain View, CA, USA)

### 3.6. Transfer Learning Model

One of the important points to consider when applying the proposed methodology to actual manufacturing is minimizing the acquisition cost of the dataset and prediction performance. If a large amount of data is required each time the model is trained for a new material, it is difficult to use as a manufacturing tool because of the high cost. Hence, a transfer learning model was developed here to reduce the training cost. The transfer learning model can be used with a smaller dataset than the model trained with the initial dataset and is a powerful tool for actual manufacturing applications [28,29]. The architecture of the transfer learning model used for the ABS material is shown in Table 5. This transfer learning model was based on the model trained with the dataset of the HDPE material. Based on earlier results on the advantages of transferring the information in the upper layer (toward the input layer side) in the prior object to the transfer model for the posterior object [30], the weights of the upper three layers were frozen, and another layer was newly added at the back when training the transfer learning model. Therefore, only the lower two layers were trained with the ABS dataset. To evaluate the efficiency, the transfer learning model and another model trained from scratch were compared. The prediction performances of both methods were compared for proportional amounts of data required to train the machine learning model without transfer learning from 90% to 10%.

## 4. Results and Discussion

### 4.1. Melt Temperature Prediction

In the melt temperature prediction process by the machine learning model in this work, the prediction accuracy was evaluated in terms of the loss, root mean-squared error (RMSE), and coefficient of determination (*R*^2^). The definitions of loss and *R*^2^ are shown in Equation (2):

(2) $$\mathrm{RMSE}=\sqrt{\sum \frac{{\left(y_{pred}-y_{ref}\right)}^{2}}{N}},\;R^{2}=1-\frac{\sum {\left(y_{i}-{\hat{y}}_{i}\right)}^{2}}{\sum {\left(y_{i}-\bar{y}\right)}^{2}}$$

In the equation, *y_pred_*, *y_ref_*, and *N* represent the temperature predicted by the model, the actual temperature, and the number of evaluation points, respectively. *R*^2^ indicates the proportion of the variance of the dependent variable that is predicted using the independent variable. $y_{i}$, $\bar{y}$, and ${\hat{y}}_{i}$ are the true value, the mean of the true values, and the predicted value, respectively. The RMSE and *R*^2^ after training completion for the three models are listed in Table 6. Models 2 and 3 show loss values of 0.1744 and 0.0718 compared with 0.2477 for Model 1, which are 29.6% and 71.0% lower than that of Model 1, respectively.

**Figure 8:**
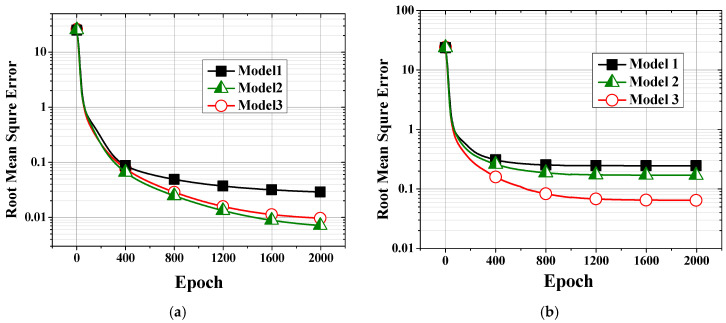
Losses (RMSEs) of the (**a**) training and (**b**) validation datasets.

**Figure 9:**
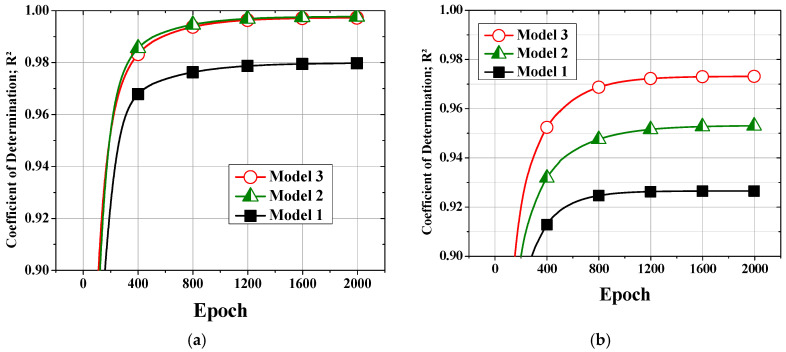
*R*^2^ values of the (**a**) training and (**b**) validation datasets.

The loss values are plotted along the epochs of the machine learning model for the training and validation datasets in Figure 8, and the corresponding *R*^2^ values are shown in Figure 9. As training proceeds, the loss continuously decreases in all cases. Models 2 and 3 proposed in this work both showed lower losses and higher *R*^2^ values than Model 1, which considered only the process setting parameters. Model 3 showed a similar or lower performance than Model 2 for training, as seen in Figure 8a and Figure 9a. However, Model 3 clearly showed a higher performance than Model 2 during validation, as seen in Figure 8b and Figure 9b. One of the reasons for these phenomena could be the ill-conditioned training and validation samples. To assess this possibility, 30 datasets of training and validation were created by randomly shuffling the dataset before processing. The 30 results were statistically processed to obtain the results in Figure 8 and Figure 9. Therefore, the possibility of ill-conditioned samples is ruled out. It is thus concluded that Model 3 represents the characteristics of the plasticizing process better than Model 2.

Model 2 shows a better performance than Model 1. However, Model 2 is limited by insufficient information for predicting the energy transferred to the material, i.e., $E_{Material}$ in Equation (1). As stated before, the magnitude of the heat loss, $E_{heat\_loss}$, could not be measured exactly and was estimated from the other terms in Equation (1) that were measured with relatively higher accuracy. During training, the insufficient information regarding the material characteristics would result in a lower prediction performance than Model 2 than Model 3, which includes these material characteristics.

**Figure 10:**
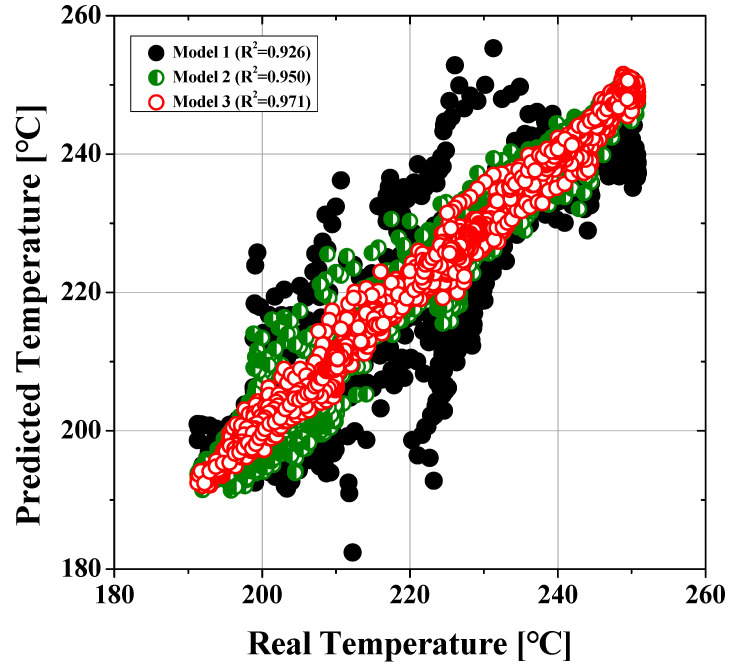
Comparisons of the prediction accuracies of the three models.

**Figure 11:**
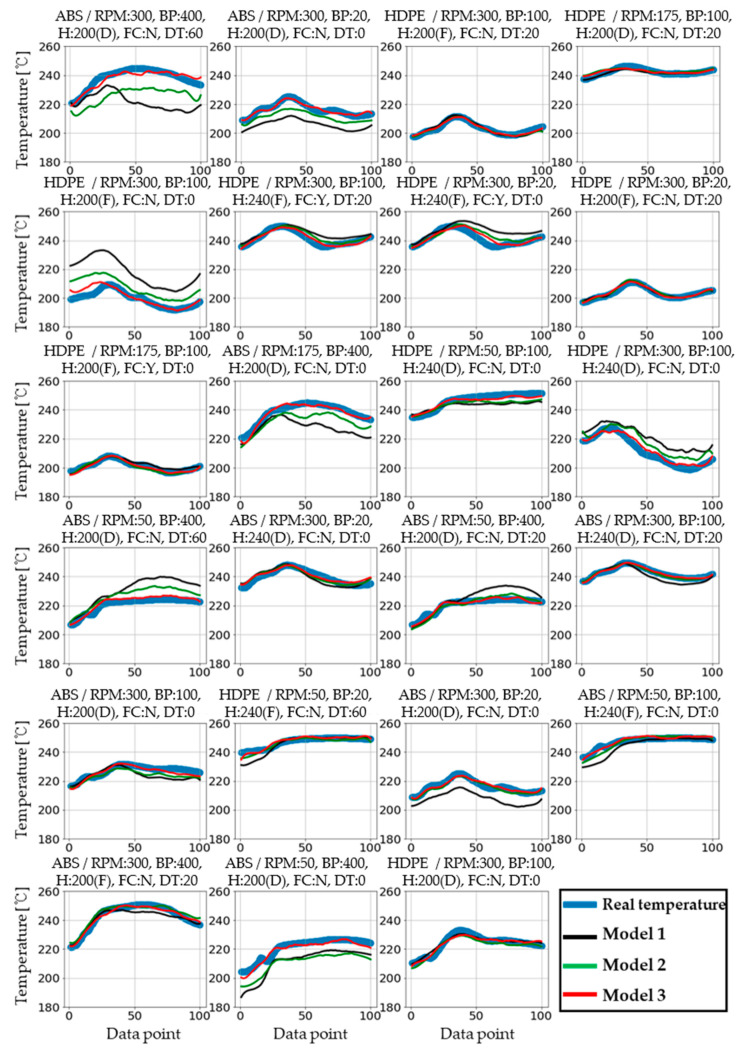
Temperature data for 23 randomly selected values and temperature prediction graphs of the three models. The horizontal axis represents the temperature points. Physically, this refers to the relative distance from the nozzle in the barrel chamber. (Plot title: material/screw RPM, back pressure in bar, heater temperature in °C (heater profile D: decrease, F: flat), forced cooling (Y/N), dwell time in s).

Figure 10 shows plots of the prediction accuracies of arbitrarily chosen validation data for the three models after training completion. Figure 11 shows the actual and predicted melt temperature profiles by the three machine learning models under randomly chosen conditions. The horizontal axes represent 100 temperature points, which are the relative distances from the nozzle. Model 3 is seen to predict the actual melt temperatures with high accuracies in most cases, whereas Model 1 shows large deviations from the actual values in some cases.

### 4.2. Improving Prediction Efficiency through Transfer Learning

**Figure 12:**
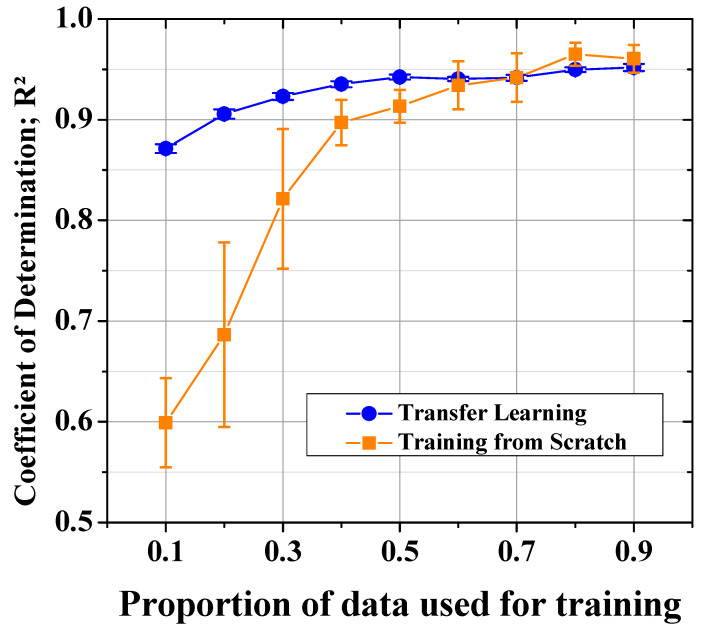
*R*^2^ values of the models with and without transfer learning.

Figure 12 shows the *R*^2^ results of the transfer learning model and the normal model trained from scratch. The architecture of both models is based on Model 3. The model trained from scratch showed a low prediction performance until sufficient data was supplied, but the transfer learning model showed a relatively high performance even with a small amount of data. In particular, the transfer learning model showed a small standard deviation over the entire dataset. The model trained from scratch produced an *R*^2^ of 0.6 with six data points, i.e., about 10% of the total dataset, which was not enough for prediction performance. However, the transfer learning model resulted in an *R*^2^ of 0.87 for the same number of data points. Both models showed similar levels of performance with at least 60% of the datasets. Nevertheless, the standard deviations of the transfer learning model were less than one tenth those of the model trained from scratch.

These results show that the transfer learning model allows the benefit of a reduction in dataset production cost and an enhancement of the prediction performance by improving the dataset efficiency and prediction reliability. In the context of this research, the time to acquire a single dataset was about an hour, including condition stabilizing and repeated experiments. However, depending on the process conditions or materials, the time required would be more than an hour, which increases data production time and enlarges the necessary dataset amount. Hence, it is appropriate to adopt the transfer learning process for industrial applications in which the cost and time are important.

However, in the case in which the input window of learning changes significantly, it is unclear whether it will guarantee a high level of transfer learning efficiency as in this study. This uncertainty can be equally considered even when not only the material but also the specifications or operation environment of the injection molding machine change significantly. It is estimated that the methodology proposed in this study does not significantly reduce the effectiveness of transfer learning, because it includes machine–material interactions such, as screw energy consumption and heater energy consumption, as well as process variables. This will be verified through further research. Further, according to previous researchers, the effectiveness of transfer learning does not completely disappear even if the material is changed [31]. Although there are differences in the properties of the two materials used in this study, the ranges of process settings parameters are not significantly different. Since the two materials have a similar process range, the weights of the three layers on the input layer side were frozen based on this similarity, and the two layers on the output side, including one newly added layer, were trained. If the process ranges of the input data to be used for pre-training and transfer learning are significantly different, it is assumed that the method of gradually using the weights of the frozen input layer for learning is effective. Additional research will be conducted to verify this assumption as a future work.

### 4.3. Contributions of Features to Temperature Prediction

**Figure 13:**
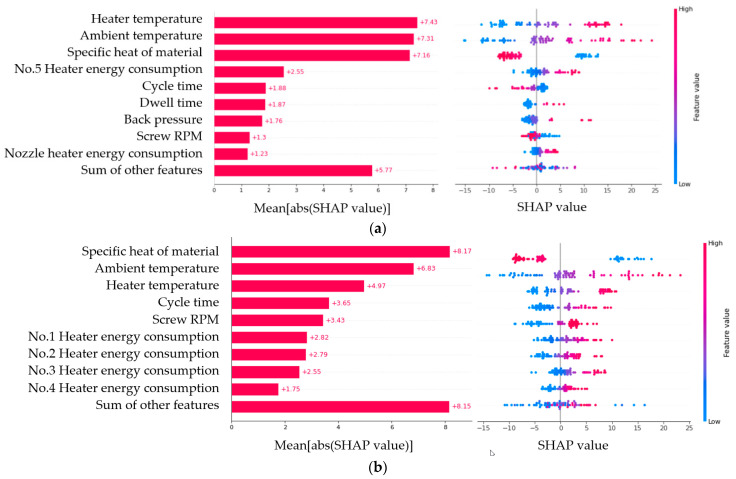

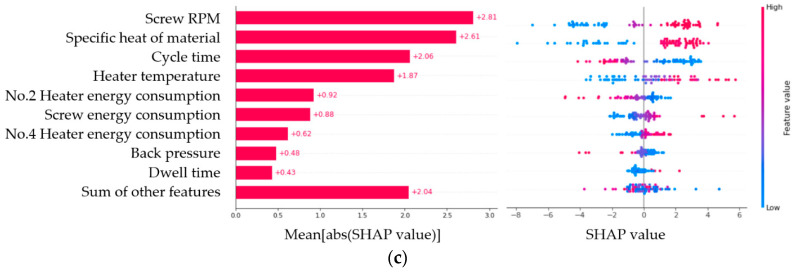
SHAP values for the (**a**) initial, (**b**) maximum, and (**c**) max–min difference temperature predictions.

Although Model 3 inductively proved to be the best method among the three models in the machine learning study, SHAP analysis was conducted to additionally verify the model performance [32]. The SHAP analysis based on the Shapley values of game theory is an interpretation method for how the machine learning model works and the contributions of each of the input features. Figure 13 shows the SHAP values of the predictions of the initial, maximum, and max–min difference temperatures in the melt temperature profile. The no. 1 heater is the barrel heater on the hopper side, and the no. 5 heater is on the nozzle side. In both cases, the features proposed in addition to the process setting parameters, such as the environmental temperature, the energy supplied to the heaters, and the specific heat as a material property, show high SHAP values. This means that the features have high correlations with the melt temperature formation process and significantly contribute to the predictions of the machine learning model.

## 5. Conclusions

For stable injection molding, the melt temperature in the barrel is an important criterion, and various plasticizing conditions affect this temperature. It has been reported that the melt temperature is not the same as the band heater temperature, and that the melt temperature in the barrel fluctuates on the basis of the plasticizing condition. There are limitations to developing fast-response temperature sensors applicable to the injection molding environment. Hence, it is necessary to find better and easier methods of predicting melt temperatures if measurements in the barrel are difficult in practice. Consequently, this work proposed a method for melt temperature prediction by a machine learning model.

To supply the labeled melt temperature data during supervised learning, a high-performance temperature sensor was developed to measure the melt temperatures. This sensor was designed to achieve a high response speed and accuracy, with adequate durability for the high-temperature and high-pressure conditions of injection molding. However, it is difficult to apply a sensitive sensor in actual mass production because of disadvantages, such as an increase in process cost, unexpected defects, and installation difficulties. Therefore, the melt temperature sensor developed herein was used for measurements in the dataset production stage for machine learning. The measured melt temperatures under various plasticizing conditions were supplied to the developed machine learning models.

To build a machine learning model for predicting melt temperatures, key features in the plasticizing process were selected from the energy flow transferred to the material by the injection molding machine, such as energy consumption of the plasticizing motor and barrel heaters. In addition, energy-related physical properties such as the specific heat of a material and part weights were included in the dataset. Thus, a more specific melt formation process was reflected in the learning compared with previous machine learning models that used only general process setting parameters.

For comparison, a model accepting only process setting parameters and a model including process data measured based on energy flow were constructed. The model proposed in this work showed the highest prediction performance. This was a logical result, because this novel model reflected the most aspects that affected the plasticizing process, and the other models lacked sufficient information.

In industrial applications, it is necessary to reduce the time and cost of dataset production. It would not be appropriate to train the machine learning model with a large amount of new datasets whenever the injection molding machines or materials are changed. For new cases, it is beneficial to exploit previously trained machine learning models; thus, a transfer learning model was built for this purpose in the present work. In comparison tests between the model trained from scratch and the transfer learning model, the transfer learning model provided a higher prediction performance with a small dataset. Thus, the transfer learning model is expected to conserve the time and cost of dataset production for machine learning in industrial applications in which these two factors are critical.

The features used in the machine learning models in this work were verified by SHAP analysis. The results showed that the energy-based features proposed in this study had a sufficient contribution. In future works, the proposed method may be expanded further to predict process data, such as cavity pressure and nozzle pressure, in addition to the melt temperature.

## Figures and Tables

**Table 1:** Experimental conditions using HDPE.

Factor	Level		
	1	2	3
Screw rotation speed (RPM)	50	175	300
Backpressure (bar)	20	100	400
Heater temperature (°C)	200	240	-
Heater profile	Flat	Decrease	-
Forced cooling	0	1	-
Dwell time (s)	0	20	60

**Table 2:** Experimental conditions using ABS.

Factor	Level	
	1	2
Screw rotation speed (RPM)	50	300
Backpressure (bar)	20	400
Heater temperature (°C)	200	240
Heater profile	Flat	Decrease
Forced cooling	0	1
Dwell time (s)	0	60

All factors were reduced to level 2.

**Table 3:** Machine learning model architecture.

Parameter	Value
Number of hidden layers	4
Number of input layer neurons	16
Number of hidden layer neurons	150
Number of output layer neurons	100
Hidden layer activation function	ReLU
Optimizer	Adamax
Loss function	RMSE
Training iterations (epochs)	1000

**Table 4:** Input features of each model.

Model	Input Features
Model 1 (conventional; process setting parameters only)	Screw rotation speed, back pressure, feed stroke, barrel heater temperatures, dwell time
Model 2 (model 1 + monitoring data)	Energy consumption of each heater, energy consumption of plasticizing motor (screw rotation), cycle time, ambient temperature
Model 3 (model 2 + material data)	Specific heat of material, product weights

**Table 5:** Transfer learning model architecture.

Parameter	Value
Number of non-trainable (frozen) layers	3 (input layer side)
Number of trainable layers	2 (1 existing layer, 1 additional layer)
Amount of data used for training (%)	10, 20, 30, 40, 50, 60, 70, 80, 90

This is used for the ABS transfer learning model from the HDPE model trained with the architecture in Table 3.

**Table 6:** Loss and *R*^2^ values after training completion of each model.

Model	RMSE	*R* ^2^
Model 1 (conventional; process setting parameters only)	0.2468	0.926
Model 2 (model 1 + monitoring data)	0.1704	0.950
Model 3 (full data; model 2 + material data)	0.0647	0.971

## Data Availability

The data presented in this study are available on request from the corresponding author.
